# Circular RNA CUL2 regulates the development of colorectal cancer by modulating apoptosis and autophagy via miR-208a-3p/PPP6C

**DOI:** 10.18632/aging.203827

**Published:** 2022-01-13

**Authors:** Bin-Lin Yang, Guo-Qiang Liu, Ping Li, Xiao-Hui Li

**Affiliations:** 1 Department of Gastrointestinal Surgery and Anal Diseases, Affiliated Hospital of Weifang Medical College, Weifang, Shandong Province, China; 2 Basic Medicine Department, Weifang Nursing Vocational College, Weifang, Shandong Province, China

**Keywords:** colorectal cancer, circular RNA CUL2, apoptosis, autophagy

## Abstract

Aim: To explore the function of circular RNA CUL2 (circCUL2) in colorectal cancer progression.

Method: RT-PCR was carried out to detect the expression of circCUL2 in colorectal cancer tissues and cell lines. Western blot and immunofluorescence were used to determine the level of autophagy. CCK-8, clone formation assay, and EdU staining were used to assess the proliferation ability. Luciferase assay verified the relationship between miR-208a-3p and circCUL2 /PPP6C. The xenograft mouse model was used to confirm the function of circCUL2 *in vivo*.

Results: The expression level of circCUL2 was down-regulated in colorectal cancer tissues and cell lines. Forcing expression of circCUL2 inhibited proliferation ability, induced apoptosis, and autophagy in colorectal cancer cells. Luciferase assay verified that miR-208a-3p could bind with circCUL2/PPP6C. Overexpression of circCUL2 could inhibit cancer progression via targeting the miR-208a-3p/PPP6C signal pathway.

Conclusion: CircCUL2 participates in progression via the miR-208a-3p/PPP6C axis in colorectal cancer. CircCUL2 would be an underlying target for the diagnosis and therapy of colorectal cancer.

## INTRODUCTION

Colorectal cancer (CRC) is the third most common cancer in the world and the fourth leading cause of cancer death in the world [1, 2]. In terms of its incidence, it accounts for almost 10% of a global cancer diagnosis. It ranks third in males and second in females. There is a significant difference in incidence between developed and developing countries. Many patients in developed countries can be diagnosed early in the disease. But there is a higher mortality rate among patients diagnosed in developing areas [3]. CRC has a high incidence in a large number of people who are sedentary and high-fat diet [4], so it is also considered to be a lifestyle-related disease. In view of the high morbidity, high mortality, and low cure rate of CRC, it is of high research value to explore the effective therapeutic targets of CRC.

With the deepening of the research, people gradually begin to pay attention to the role of non-coding-RNA in the progression of cancer [5, 6]. Circular RNA (circRNA) is a kind of high abundance non-coding covalent closed RNA formed by exon sequence and intron sequence [7, 8]. They can be used as real miRNA sponges to regulate gene expression, and they can also be selectively spliced or act as transcription factors to encode proteins [9, 10]. Many studies have shown that a variety of circRNA is abnormally expressed in colorectal cancer, and its abnormal expression is related to the proliferation, apoptosis, invasion, and other biological functions of colorectal cancer cells [11]. Many studies have found that many circRNA is associated with the proliferation of colorectal cancer cells. Meanwhile, it is also found that circRNA could participate in the regulation as ceRNA. In the study of 60 pairs of patient tissue samples, Li et al. found that the expression of circVAPA was upregulated in tissues and was related to the proliferation of CRC cells. Then, they verified the relationship between up-regulation of circVAPA and CRC through *in vivo* and *in vitro* experiments and found that cireVAPA can promote the proliferation of colorectal cancer cells by adhering to miR-101 [12]. Jin et al. screened out the down-regulated circRNA, hsa_circ_0000523, in colorectal cancer and found that it could promote cell proliferation by siRNA detection. Subsequently, the authors found that hsa_circ_0000523 can promote the apoptosis of colorectal cancer cells by acting on miR-31 and activating Wnt/β-catenin signal pathway [13]. Min et al. found that overexpression of circRNA_104916 can significantly inhibit the migration and invasion of tumor cells by inhibiting epithelial-interstitial transformation [14]. Li et al. found that circITGA7 can regulate the metastasis of colorectal cancer cells by regulating the Ras pathway and down-regulating the ITGA7 gene [15].

In previous research, it was found that circCUL2 could prevent tumor growth and control cisplatin sensitivity via miR-142-3p/ROCK2-mediated autophagy induction in gastric cancer. However, there was no research on circCUL2 in CRC. Here, we found the downregulated expression level of circCUL2 in CRC tissues and cells. Forced expression of circCUL2 inhibited proliferation on CRC cells. CircCUL2 could special bind with miR-208a-3p and regulate PPP6C, the downstream target of miR-208a-3p, via promoting apoptosis and autophagy, which could be a new therapeutic target of CRC.

## RESULTS

### The decreased level of circCUL2 in CRC could bind with miR-208a-3p

**Figure 1:**
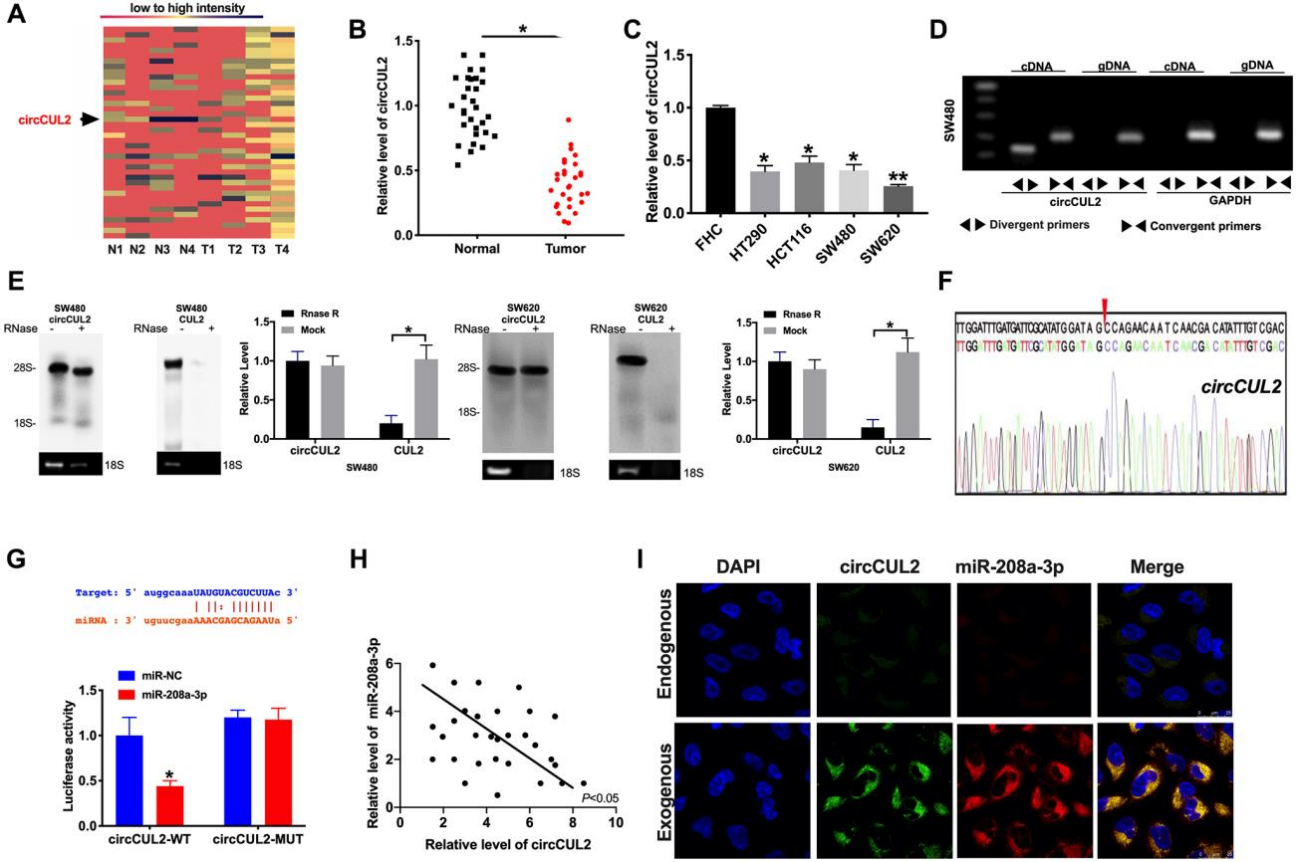
**circCUL2 is downregulated in CRC tissues and cells.** (**A**) The chip assay was performed in CRC tumor tissues and paired adjacent normal tissues. (*n* = 4). (**B**) The expression level of circCUL2 was detected in tumor tissues and paired adjacent normal tissues. *n* = 30. ^*^P < 0.05. (**C**) The expression level of circCUL2 in the CRC cell line, FHC cell lines was indicated as control. *n* = 5. ^*^P < 0.05, ^**^P < 0.01. (**D**) The PCR products of circCUL2 and linear CUL2 were tested by gel electrophoresis. Divergent primers amplified circCUL2 in cDNA but not genomic DNA (gDNA). Convergent primers amplified linear CUL2 in both cDNA and gDNA. GAPDH was used as a linear control. (**E**) CircCUL2 was resistant to RNaseR digestion in SW480 and SW620 CRC cells. *n* = 3. ^*^P < 0.05. (**F**) The result of Sanger sequencing. (**G**) The binding sites between circCUL2 and miR-208a-3p (upper), luciferase assay report verified the relationship between circCUL2 and miR-208a-3p (lower). *n* = 4. ^*^P < 0.05. (**H**) The correlation analysis between circCUL2 and miR-208a-3p in 30 paired CRC tissues. *n* = 30. ^*^P < 0.05. (**I**) Co-localization between circCUL2 and miR-208a-3p was revealed by fluorescence *in situ* hybridization.

Microarray analysis was performed on CRC tumor tissues and adjacent normal tissues. CircCUL2 was found down-regulated in CRC tumor tissues (Figure 1A). Then we collected CRC tumor tissues and adjacent normal tissues to evaluate the expression level of circCUL2. Compared with adjacent normal tissues, the expression level of circCUL2 was down-regulated in CRC tumor tissues (Figure 1B). Then we cultured CRC cell lines (HT290, HCT116, SW480, SW620), qRT-PCR assay results performed the down-regulated circCUL2 level in CRC cell lines compared with FHC cells (Figure 1C). We first designed two sets of primers for circCUL2: convergent primers that were expected to amplify only the linear form, CUL2 mRNA, and divergent primers to amplify only the circular form, circCUL2. cDNA and genomic DNA (gDNA) were used as templates, and the specificity of the qRT-PCR was validated by 1% agarose gel electrophoresis. As expected, the single and distinct product of the expected size was amplified using the divergent primers in only cDNA, while no product was amplified with the divergent primers in gDNA (Figure 1D). RNase R digestion was conducted to verify the circular characteristics of circCUL2. The data showed that circCUL2 was resistant to RNase R digestion (Figure 1E). Through sanger sequencing, we confirmed that the circRNA sequence amplified by the primer was identical to its sequence in circbase (Figure 1F).

### MiR-208a-3p would be a target of circCUL2

Bioinformatics site forecasted that miR-208a-3p was an underlying target of circCUL2. Luciferase assay verified the relationship of miR-208a-3p and circCUL2. The decreased luciferase activity was observed in miR-208a-3p mimics and circCUL2 *wild type* (circCUL2-WT) co-transfection group, while not in miR-208a-3p mimics and circCUL2 *mutant* (circCUL2-MUT) co-transfection group (Figure 1G). Further, we found a negative relationship between miR-208a-3p and circCUL2 in CRC tumor tissues (Figure 1H). Therefore, the relationship of circCUL2 and miR-208a-3p was verified by FISH assay, and we found that miR-208a-3p was co-localized with circCUL2 in the cytoplasm, which once performed a relationship between circCUL2 and miR-208a-3p (Figure 1I).

### circCUL2 prevents tumor progression of CRC via targeting miR-208a-3p *in vitro*

**Figure 2:**
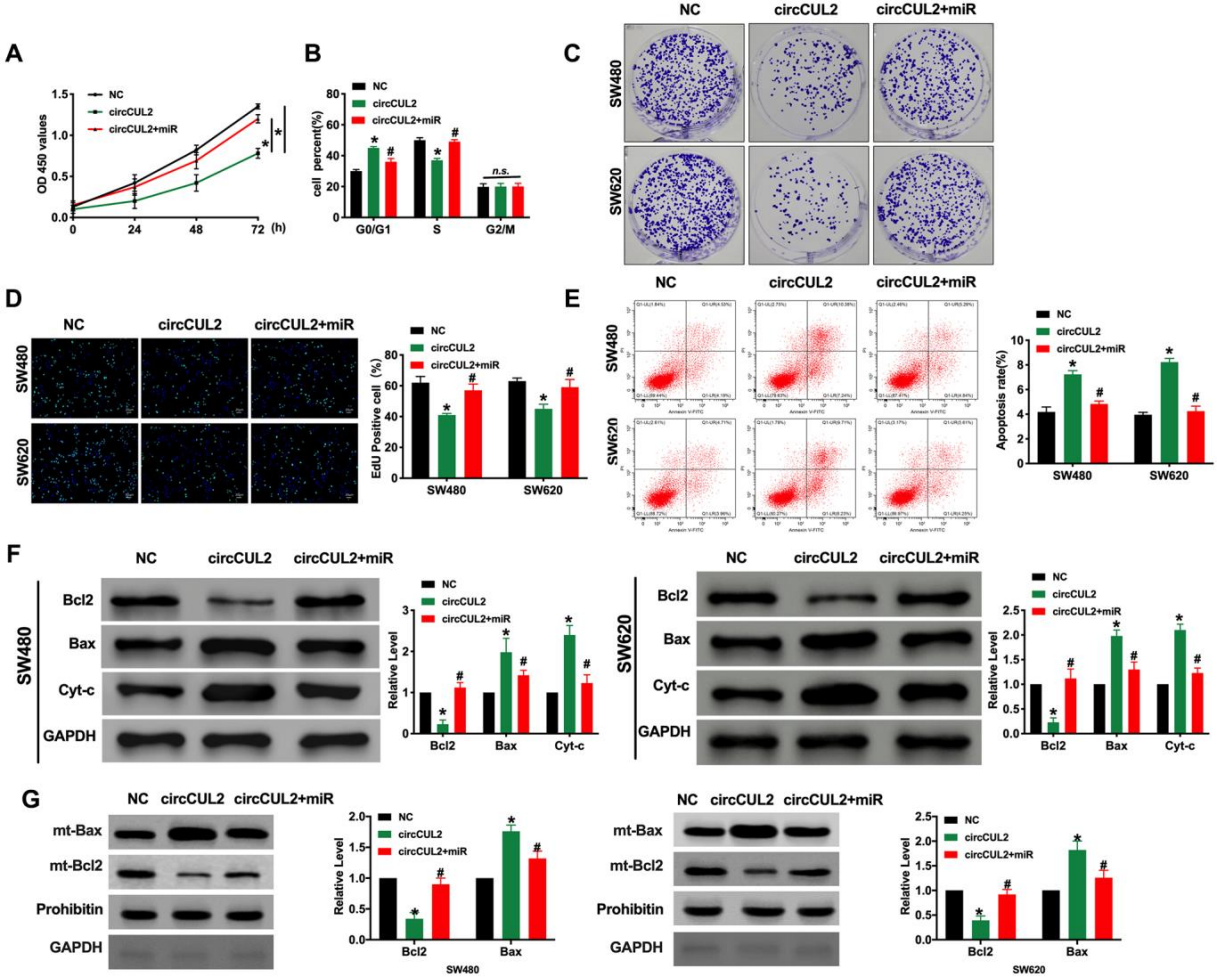
**circCUL2 inhibits tumor progression via targeting miR-208a-3p.** (**A**) CCK-8 assay was performed to detect the cell viability in CRC cells. *n* = 6. ^*^P < 0.05. (**B**) Cell cycle was determined in CRC cells by flow cytometry. *n* = 5. ^*^P < 0.05 vs. NC group, ^#^*P* < 0.05 vs. circCUL2 group. *n.s.* indicated no significance. (**C**) The clone formation assay was used to assess the cell proliferation. (**D**) The EdU assay results in CRC cells. *n* = 5. ^*^P <0.05 vs. NC group, ^#^*P* < 0.05 vs. circCUL2 group. (**E**) The flow cytometry was performed to detect the apoptosis level in CRC cells. *n* = 5. ^*^P < 0.05 vs. NC group, ^#^*P* < 0.05 vs. circCUL2 group. (**F**) The apoptosis-associated protein Bcl2, Bax, and Cytochrome-c (Cyt-c) levels were detected in CRC cells. *n* = 5. ^*^P < 0.05 vs. NC group, ^#^*P* < 0.05 vs. circCUL2 group. (**G**) Mitochondrial-Bcl2 and Bax levels was detected in CRC cells. *n* = 4. ^*^P < 0.05 vs. NC group, ^#^*P* < 0.05 vs. circCUL2 group.

To explore the function of circCUL2 in CRC progression, we constructed the plasmid for overexpression of circCUL2 in CRC cell lines. CCK-8 assay was confirmed that circCUL2 inhibited cell viability in CRC cell lines which were prevented by overexpression of miR-208a-3p (Figure 2A). Further, cell cycle assay determined that circCUL2 plasmid could prevent CRC cells from G0/G1 phase entering into S phase, while miR-208a-3p blocked the function of circCUL2 (Figure 2B). Clone formation experiments performed that circCUL2 inhibited clone formation in CRC cells, forced expression of miR-208a-3p abolished the role of circCUL2 on clone formation (Figure 2C). Meanwhile, circCUL2 attenuated the proliferation of CRC cells via EdU assay layout, miR-208a-3p mimics performed the hinder function on overexpression of circCUL2 (Figure 2D). In summary, circCUL2 would regulate the proliferation ability of SW480 and SW620 cells via targeting miR-208a-3p. Next, we performed a flow cytometry assay and western blot to assess the apoptosis level in CRC cells after circCUL2 and miR-208a-3p transfection. We observed that circCUL2 induced apoptosis in CRC cells, which was prevented by overexpression of miR-208a-3p (Figure 2E and 2F). Further, the mitochondrial Bax and Bcl2 level also verified that circCUL2 induced apoptosis in CRC cells (Figure 2G).

### circCUL2 induces autophagy in CRC via targeting miR-208a-3p *in vitro*

**Figure 3:**
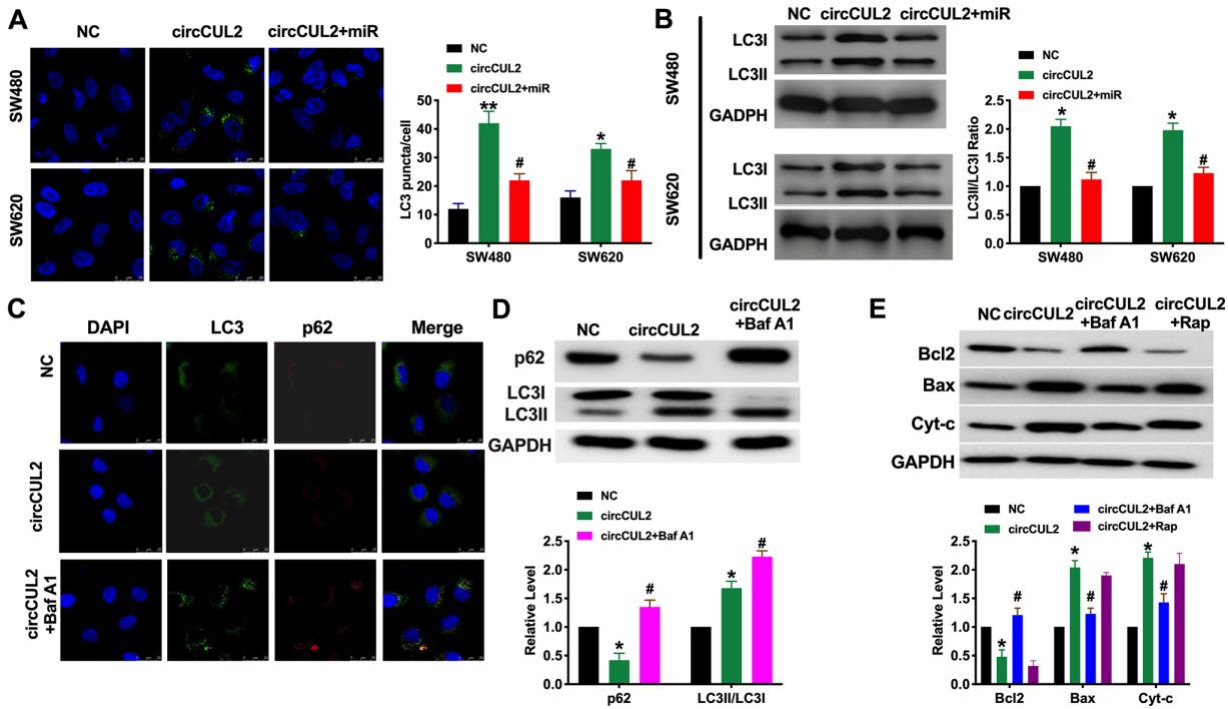
**circCUL2 induces autophagy level in CRC cells via regulating miR-208a-3p.** (**A**) The LC3 level was detected by immunofluorescence. *n* = 5. ^*^P < 0.05, ^**^P < 0.01 vs. NC group, ^#^*P* < 0.05 vs. circCUL2 group. (**B**) The protein level of LC3 in CRC cells. *n* = 5. ^*^P < 0.05 vs. NC group, ^#^*P* < 0.05 vs. circCUL2 group. (**C**) SW480 cells were treated with circCUL2 and Bafilomycin A1(Baf A1). LC3 and p62 puncta were measured by immunofluorescence analysis. (**D**) Immunoblotting analysis of protein levels of the SW480 cells treated for 100 nM Baf A1 for 24 h. *n* = 3, ^*^P < 0.05 vs. NC group, ^#^*P* < 0.05 vs. circCUL2 group. (**E**) The apoptosis level of SW480 cell after autophagy inhibitor (Baf A1, 100 nM, 24 h) and inducer (Rapamycin, Rap, 100 nM, 24 h). NC group, ^#^*P* < 0.05 vs. circCUL2 group.

Autophagy could be both activated and inhibited in colorectal cancer. It plays a double-edged role in the occurrence and development of colorectal cancer. Autophagy could inhibit tumors in the early stage of cancer, while autophagy in the late stage contributes to the survival of tumor cells in an adverse environment. Some autophagy-related protein, LC3, has become an indicator of prognosis in patients with colorectal cancer. Autophagy is a dynamic process. In our research, we found that circCUL2 promoted autophagy level, which was indicated by increased LC3 fluorescence spot and protein level. However, forced expression of miR-208a-3p prevented autophagy in CRC cells (Figure 3A and 3B). After treated with Bafilomycin A1(Baf A1, 100nM) for 24 h, the Inhibition of autophagosome and lysosomal binding leads to LC3II accumulation while restoring p62 levels (Figure 3C and 3D). Further, Baf A1 treatment prevented the function of circCUL2 on apoptosis level. While Rapamycin co-treated with circCUL2 did not ggravate apoptosis level (Figure 3E).

### PPP6C is downstream of miR-208a-3p

**Figure 4:**
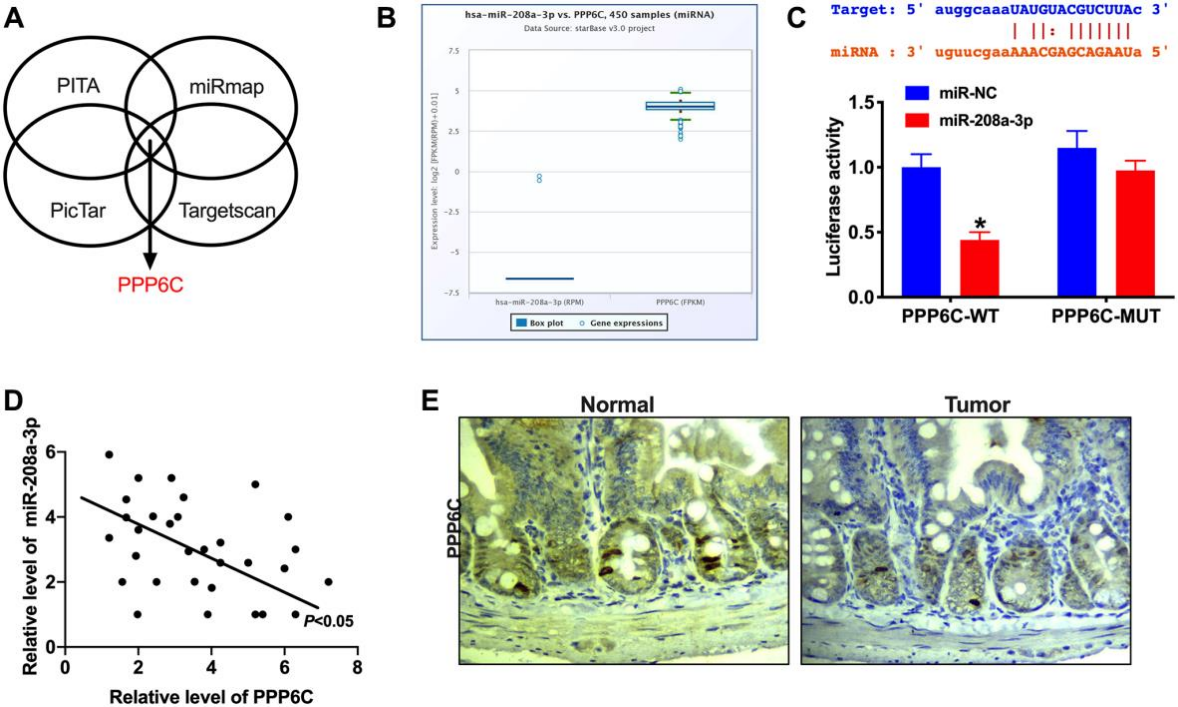
**PPP6C is a target of miR-208a-3p.** (**A**) Bioinformatics site (PITA, miRmap, PicTar, Targetscan) forecasted that PPP6C was downstream of miR-208a-3p. (**B**) The negative relationship between miR-208a-3p and PPP6C in Starbase 3.0. (**C**) The binding sites between PPP6C and miR-208a-3p (upper), luciferase assay report verified the relationship between PPP6C and miR-208a-3p (lower). *n* = 4. ^*^P < 0.05. (**D**) The correlation analysis between PPP6C and miR-208a-3p in 30 paired CRC tissues. *n* = 30. ^*^P < 0.05. (**E**) IHC staining was performed to detect the level of PPP6C in human CRC tumor tissue and adjacent normal tissues.

MicroRNA (miRNA) could specifically recognize the corresponding target sites in the 3 ′UTR of target genes, regulate the expression of specific target genes, and protein synthesis at the post-transcriptional level, which performs a key role in cell proliferation, apoptosis, and differentiation. Four bioinformatics websites confirmed that PPP6C was an underlying target of miR-208a-3p (PITA, miRmap, PicTar, Targetscan, Figure 4A). Starbase 3.0 database showed the negative relationship between miR-208a-3p and PPP6C (Figure 4B).

Luciferase assay verified that miR-208a-3p could bind with 3′UTR of PPP6C (Figure 4C). RT-PCR assay performed the negative relationship between miR-208a-3p and PPP6C in CRC tumor tissues (Figure 4D). Meanwhile, IHC assay results observed the down-regulated expression level of PPP6C in CRC tumor tissues (Figure 4E).

**Figure 5:**
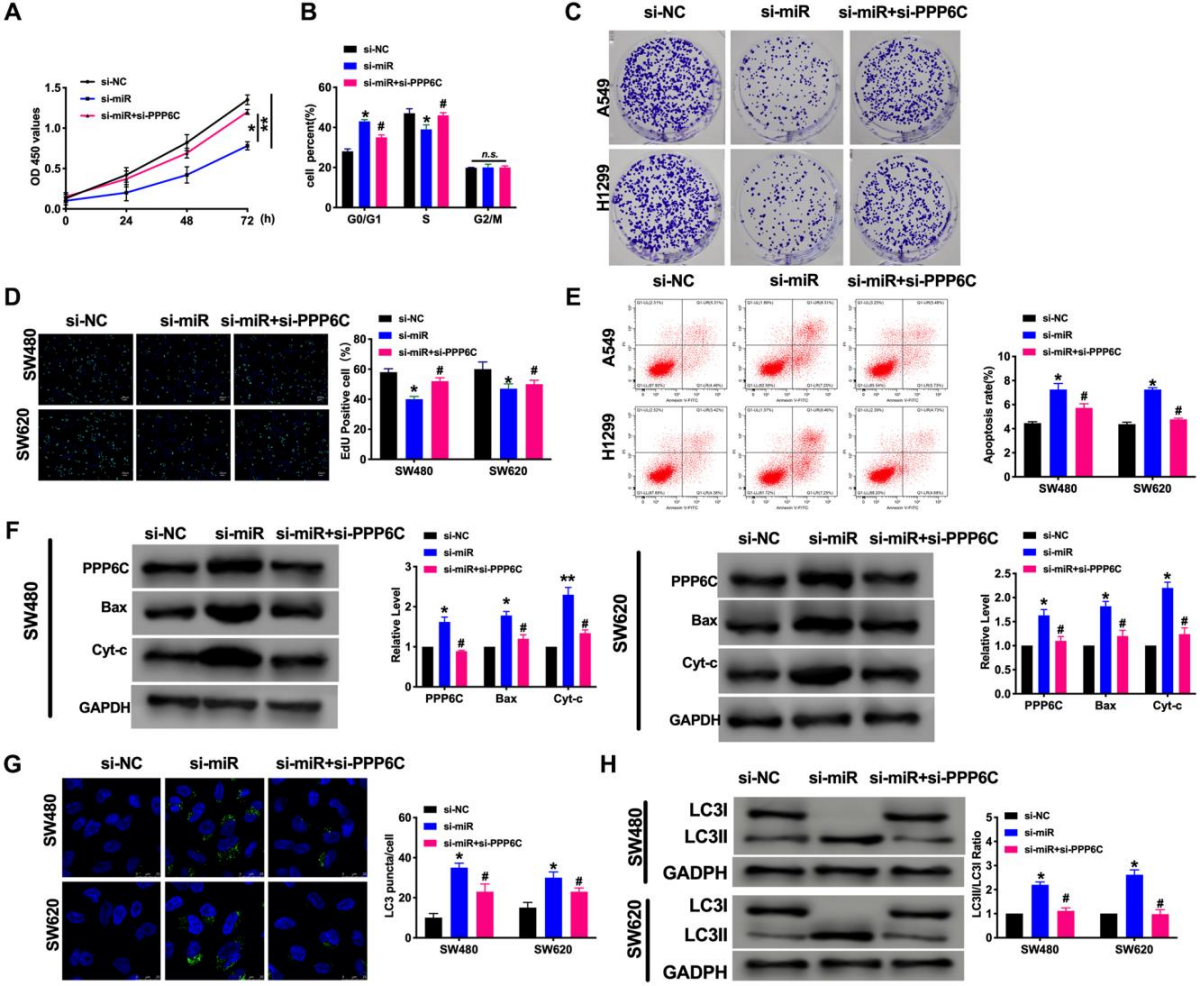
**PPP6C could be downstream of miR-208a-3p and involves in CRC tumor progression.** (**A**) CCK-8 assay was performed to detect the cell viability in CRC cells. *n* = 6. ^*^P < 0.05, ^**^P < 0.01. (**B**) Cell cycle was determined in CRC cells by flow cytometry. *n* = 5. ^*^P < 0.05 vs. si-NC group, ^#^*P* < 0.05 vs. si-miR group. *n.s.* indicated no significance. (**C**) The clone formation assay was used to assess the cell proliferation. (**D**) The EdU assay results in CRC cells. *n* = 5. ^*^P < 0.05 vs. si-NC group, ^#^*P* < 0.05 vs. si-miR group (**E**) The flow cytometry was performed to detect the apoptosis level in CRC cells. *n* = 5. ^*^P < 0.05 vs. si-NC group, ^#^*P* < 0.05 vs. si-miR group. (**F**) The apoptosis-associated protein Bcl2, Bax, and Cytochrome-c (Cyt-c) levels were detected in CRC cells. *n* = 5. ^*^P < 0.05, ^**^P < 0.01 vs. si-NC group, ^#^*P* < 0.05 vs. si-miR group. (**G**) The LC3 level was detected by immunofluorescence. *n* = 5. ^*^P < 0.05 vs. si-NC group, ^#^*P* < 0.05 vs. si-miR group. (**H**) The protein level of LC3 in CRC cells. *n* = 5. ^*^P < 0.05 vs. si-NC group, ^#^*P* < 0.05 vs. si-miR group.

Next, we explored the function of PPP6C on CRC progression. Si-miR-208a-3p and si-PPP6C were co-transfected in CRC cells, the proliferation ability was assessed by CCK-8, cell cycle, clone formation, and EdU assay. The results showed that si-miR-208a-3p prevented the proliferation in CRC cells, while co-transfected with si-PPP6C blocked the function of si-miR-208a-3p (Figure 5A–5D). Further, the apoptosis level was confirmed by flow cytometry and western blot. We found that si-miR-208a-3p could induce apoptosis in CRC cells (Figure 5E and 5F). Then we also found silencing of miR-208a-3p generated the autophagy level, which was indicated by increased LC3 fluorescence spots and protein level (Figure 5G and 5H). Taken together, PPP6C involved in CRC progression, which could be a target of miR-208a-3p.

### circCUL2 prevents tumor development *in vivo*

**Figure 6:**
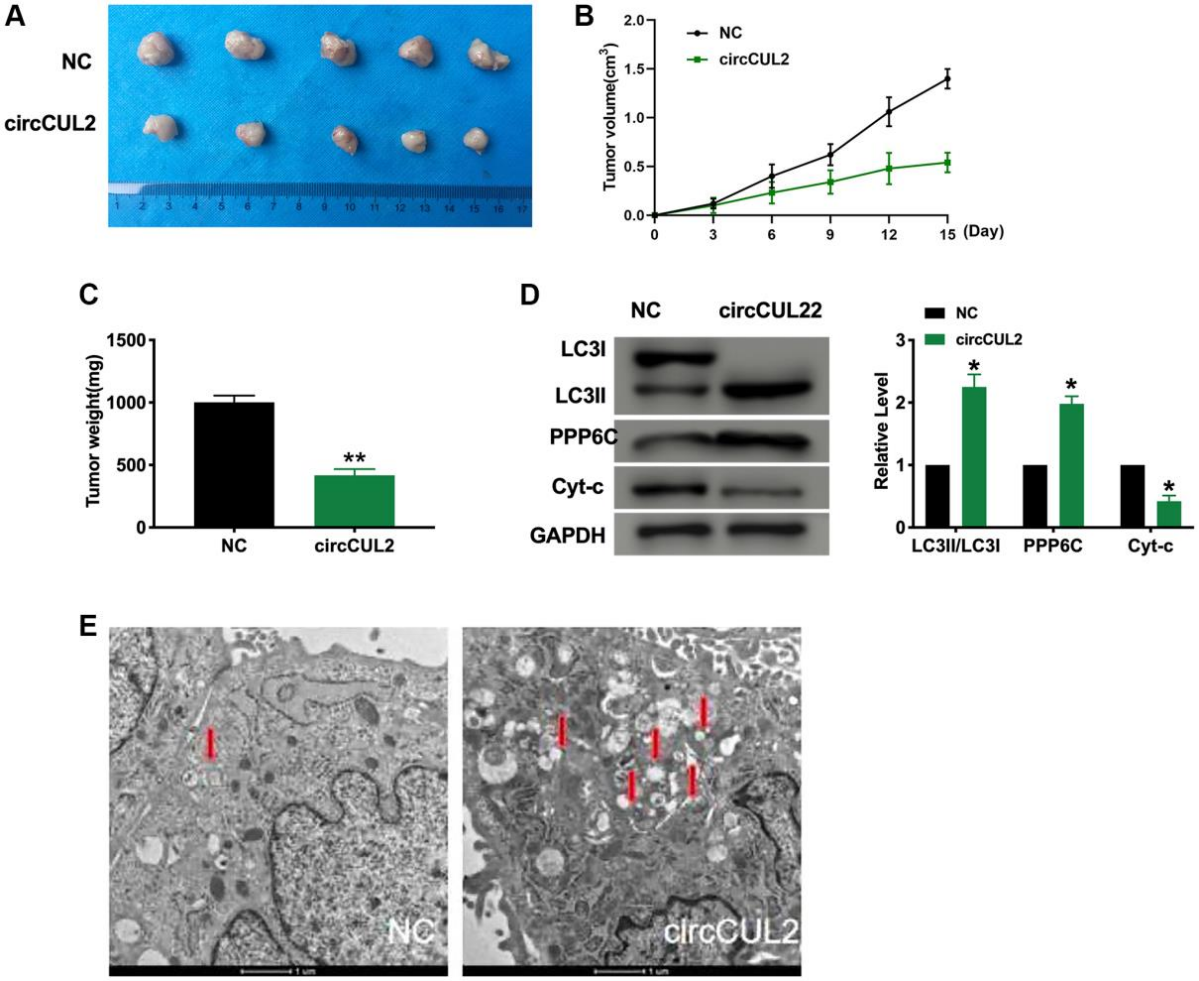
**circCUL2 prevents tumor growth *in vivo*.** (**A**) Tumors were removed from the mice 15 days after circCUL2, and NC-SW480 cells were treated, respectively *n* = 5. (**B**–**C**) Tumor volume and weight were shown after the tumors were collected. *n* = 5, ^*^P < 0.05, ^**^P < 0.01. (**D**) The protein level of LC3, PPP6C, Cyt-c in tumor tissues. *n* = 5, ^*^P < 0.05. (**E**) Transmission electron microscopy (TEM) analysis showing autophagosome (arrowed) after treated with circCUL2. The autophagosome is shown by the red arrow.

Further, Next, Xenograft assay was carried out to explore the tumor progression. circCUL2 SW480 cells/NC ASW480 cells were subcutaneously injected to the left hind limb of the mouse constructed the tumor-bearing model. The results showed that circCUL2 prevented tumor progression *in vivo* (Figure 6A–6D). We then detected the autophagy and apoptosis level by western blot. Transmission electron microscopy (TEM) analysis showing autophagosome (arrowed) after treated with circCUL2 (Figure 6E). The results showed that circCUL2 promoted autophagy and apoptosis level *in vivo*.

## DISCUSSION

At present, more and more studies have shown that circRNA is an important non-coding RNA molecule, which plays a variety of biological regulatory roles. Moreover, the more stable closed-loop structure of circRNA makes it a more potential clinical marker [16]. At present, many studies have found that plasma circRNA can be used as a molecular biomarker for the diagnosis of diseases.

Many circRNA have been found to be involved in the regulation of apoptosis of CRC cells through the mechanism of ceRNA. Zhang also found that hsa_circ_0020397 can regulate the expression of miR-138 by targeting TERT and PD-L1 and then handle the apoptosis of CRC cells [17] He et al. found that circRNA-ACAP2 can regulate the apoptosis of CRC cells through the hsa-miR-21-5p/Tiam1 signal axis [18]. Both circ_0026344 and circ_000753431 are associated with poor prognosis and can significantly restrict the apoptosis of CRC cells [19]. It has also been found that circRNA_100290 can promote the proliferation of CRC cells by targeting miR-516b [20]. CircHIPK3 can encourage the proliferation of CRC cells by absorbing miR-7 [21]. Hsa_circ_0136666 can encourage the proliferation of CRC cells through the miR-136/SH2B1 axis [22]. Many circRNAs can affect the migration and invasion of CRC cells, participate in the development of cancer, and then affect the process of cancer. Li et al. found that circDDX17 can be used as a tumor suppressor, and silencing circDDX17 can promote the proliferation and apoptosis of CRC cells as well as metastasis and invasion [23]. Hsiao et al. found that silencing circRNACCDC66 can inhibit tumor growth and tumor cell invasion [24]. Some studies have also found that hsa_circRNA_104700 is significantly associated with distal metastasis of CRC cells [25]. Jin et al. found that circRNA hsa_circ_0136666 was significantly overexpressed in colon cancer. This study shows that hsa_circ_0136666 can promote the proliferation, metastasis, and invasion of CRC cells. The researchers found that hsa_circ_0136666 can be used as a molecular sponge of miR-136 to remove the negative regulation of miR-136 on the SH2B1 gene, and the SH2B1 gene is involved in the formation of CRC. It is inferred that circRNA hsa_circ_ 0136666 regulates the occurrence and development of CRC through the miR-136-SH2B1 axis [22]. Xia et al. found that hsa_circ_0053277 was significantly overexpressed in CRC cells and held the high expression of MMP14, a proto-oncogene, by combining with miR-2467-3p to promote the proliferation, infiltration, and metastasis of tumor cells [26]. Chen et al. found that there is an M6A modification site in circNSUN2 in the nucleus of CRC, which can combine with YTHDC1 protein to produce nucleus and express in the cytoplasm. After up-regulation, it binds to IGF2BP2 protein and HMGA2 to form a triad complex, which stabilizes the expression of HMGA2 and promotes the liver metastasis of CRC cells [27].

In a previous study, circCUL2 was found downregulated in gastric cancer tissues and cell lines. It suppressed the proliferation, migration, and invasion of gastric cancer cells. Furthermore, circCUL2 was downregulated in cisplatin-resistant gastric cancer cell lines and modulated cisplatin sensitivity. circCUL2 may be involved in tumorigenesis and chemoresistance by competitively binding to miR-142-3p and by modulating ROCK2 expression and autophagy activation. We paid attention to detect the function of circCUL2 in CRC. We observed that circCUL2 was downregulated in CRC. Further, we also found that forced expression of circCUL2 could inhibit the proliferation of CRC cells by performing CCK-8, clone formation assay, cell cycle assay. Forced expression of circCUL2 induced apoptosis and autophagy activation. To detect the underlying mechanisms of circCUL2 in CRC, we used bioinformatics techniques to forecast the underlying miRNA target for circCUL2. Four bioinformatics confirmed that miR-208a-3p could bind with circCUL2. It was reported that miR-208a-3p was up-regulated in CRC tissues. Its high expression was statistically associated with distant metastasis and TNM stage. Functional assays revealed inhibition of miR-208a-3p suppressed proliferation, invasion, and migration and induced cell apoptosis of CRC cells. Protein phosphatase 6 (PP6) is a member of the phosphoprotein phosphatase (PPP) family of Ser/Thr protein phosphatases, which are conserved throughout eukaryotes. PP6 exhibits multiple roles in regulating several cellular processes, including cell cycle, autophagy, DNA damage repair, and lymphocyte development. Furthermore, we determined that PPP6C could bind with miR-208a-3p.

In previous research, autophagy and apoptosis were involved in the tumorigenesis and progression of cancer. Apoptosis is a strict pathway that controls cell death under physiological and pathological conditions and is called the therapeutic target of CRC. Autophagy is a cellular defense pathway that starves or regulates the survival and death of CRC cells [28, 29]. In previous research, circRNAs could target downstream genes via the ceRNA mechanism to trigger autophagy in CRC cells. In our results, PPP6C was markedly decreased in CRC cells, and PPP6C might involve in circCUL2/miR-208a-3p pathway to regulate autophagy and apoptosis level in CRC cells.

The occurrence and development of cancer contain complex pathophysiological processes, which can not be explained alone from a certain aspect, but circRNA may play a role in many processes of pathophysiological changes of cancer cells. Therefore, people often study the role of circRNA from many aspects, that is, to study the effect of a kind of circRNA on cancer cell proliferation, apoptosis, invasion and migration, and so on. A large number of studies have also shown that many circRNA is associated with multiple processes of colorectal cancer. With further research on the process of cancer in the future, circRNA may also have a potential role in other directions other than the currently known changes.

## CONCLUSIONS

In conclusion, our study indicated that circCUL2 was significantly down-regulated in CRC patients’ tissues and cell lines. Forced expression of circCUL2 could prevent proliferation ability, induced apoptosis, and autophagy in CRC cells. CircCUL2 prevented CRC progression by sponging miR-208a-3p and regulating PPP6C, which could serve as a potential therapeutic target for CRC treatment.

## MATERIALS AND METHODS

### Cell culture

HT290, HCT116, SW480, and SW620 human colorectal cancer cell lines, FHC normal colonic epithelial cells (all purchased from the cell bank of Shanghai Chinese Academy of Sciences). HCT116 and HT290 cells used McCoy’s 5A medium containing 10% fetal bovine serum (FBS) (BI Company), FHC, SW480, and SW620 used RPMI-1640 (HyClone) medium containing 10% (FBS). All cell lines were incubated in sterilized cell incubators (5% CO_2_, 37°C).

### Cell transfection

The plasmid was purchased from Shanghai Jikai Genome Co., Ltd. The cells were seeded in a 6-well plate according to 5 × 10^5^ cells per well. When the cells reached 90% fusion, the cells were transfected by Lipofectamine 2000 (Invitrogen Company, Shanghai) with the plasmid, miRNA mimics, or siRNA. The cells were harvested for Real-Time PCR and Western blot detection for 48 hours. MiR-208a-3p mimics, NC mimics, and miR-208a-3p and NC inhibitors were synthesized by Suzhou GenePharma Co., Ltd. The sequences used were as follows: miR-208a-3p mimics, 5′-AUA AGACGAGCA A A A AGCU UGU-3′ and 5′-AGCUUUUUGCUCGUCUUAUUU-3′; siRNA miR-208a-3p, 5′-ACA AGCUUUUUGCUCGUCU UAU-3′; miRNA NC, 5′-UUCUCCGA ACGUGUCACGUTT-3′ and 5′-ACGUGACACGUUC GGAGAATT-3′ and siRNA NC, 5′-CAGUACUUU UGUGUAGUACA A-3′. The siRNA PPP6C sequences: 5′-GATCCGCCAAAGTTATTCCGAGCA GTTTTCAAGAGAAACTGCTCGGAATAACTTTGGTTTTTTACGCGTG-3′; siRNA Negative control (si-NC) 5′-AATTCACGCGTAAAAAACCAAAGTTA TTCCGAGCAGTTTCTCTTGAAAACTGCTCGGAATAACTTTGGCG-3′.

### Cell cloning formation assay

The well-growing cells were digested with 0.25% trypsin and centrifuged. The cells were purged with sterile 1 × PBS 2 times and counted under light microscope with a cell counting board. There were 500 cells/well in 6-well plate, and the complete medium was cultured for 10–14 days, and the fresh medium was changed according to the state of cell growth. Discarding the medium, wash with PBS, add 1ml Giemsa A solution, stable at room temperature for 4 min, then continue to add 2ml B solution, gently mix well, stand at room temperature for 8 min. Recoding the results.

### CCK- 8 assay

The cells in the logarithmic growth phase were incubated in 96-well plate (5 × 10^3^/well). After corresponding treatment, the cells in each group were cultured for 24 hours, then added to 10% CCK-8 medium and incubated in the incubator for 2 hours. Finally, the absorbance value (OD) of each sample at 450nm wavelength was determined by an enzyme labeling instrument.

### Northern blotting

We performed Northern blotting using NorthernMax Kit (Thermo Fisher Scientific, California, USA). Briefly, RNA (15 μg for detection of endogenous circCUL2 and 8 μg for detection of linear CUL2) was denatured with 3 volumes formaldehyde load dye (Ambion) for 15 min at 65°C and loaded on 1% agarose gel. After the electrophoresis, RNA was transferred on Hybond N+membrane (GE Healthcare, Uppsala, Sweden) by capillary transfer. Transferred RNA was ultraviolet-crosslinked (at 265 nm). Pre-hybridization was performed at 68°C for 30 min and hybridization was performed at 68°C overnight. The membrane was washed with 2 × SSC 0.1% SDS twice 5 min at room temperature, then twice 15 min with 0.1 × SSC 0.1% SDS at 68°C. The membrane was hybridization with anti-DIG antibody and washed using DIG Wash and Block Buffer Set (Roche, Indianapolis, IN, USA). After washing, the blot was detected with the DIGluminescence detection kit (Roche). DIG-labeled probes were prepared using DIG Northern starter Kit (Roche) by *in vitro* transcription with PCR products as templates for T7 transcription.

### circRNA chip analysis

Four pairs of total RNA extracted from CRC and its adjacent tissues were extracted by the Trizol method, and the total RNA was purified after passing the electrophoresis quality inspection of AgilentBioanalyzer 2100 (Agilent Company, USA). The D value of RNA at the wavelength of 260 nm and 280 nm was detected by NanoDropND-2000 spectrophotometer, and the ratio of the gray value of 28S/18S RNA band was calculated by RNA electrophoresis. The quality and total amount of RNA were analyzed by RIN (RNA Integrity Number) value. According to the operating instructions provided by the expression profile microarray kit (Low Input Quick Amp Labeling Kit, One-Color of American Agilent company, the sample RNA was amplified and labeled, and the labeled RNA was purified by RNeasy Mini Kit. The labeled samples were assembled into the hybridization chamber according to the standard hybridization process and kit provided by the Agilent expression profile microarray and were hybridized at 65°C for 17 h (after the rotation speed was 10 rpm), the chip was washed with an elution kit. The hybrid chip was scanned by Agilent Microarray Scanner. Finally, the hybrid signal values are separated and standardized. The logarithmic multiple of difference (fold change, FC) was taken as the logarithm value, and the adjusted log2FC > 2 and *P* < 0.05. Cluster analysis was carried out according to the expression value of circRNA in each sample, and a heat map was drawn to show the expression of circRNA in different samples.

### Real-Time PCR

Total RNA was extracted by TRIzol method as a template, and reverse transcription for cDNA; experimental flow refers to the operating instructions provided by GoScriptTM reverse transcription kit; reaction conditions: first, random primers and cDNA were mixed, then incubated at 70°C for 5 min, then quickly cooled on ice for 5 min, buffer, reverse transcriptase, nucleotides, and magnesium ions were added, and annealed at 25°C for 15 min, 42°C to extend 60min. Then the target gene was amplified according to the operation flow of the SYBRGreen method provided by real-time fluorescence quantitative PCR kit. The reaction conditions were as follows: pre-denatured 95°C 5 min, 95°C denatured 1min, 60°C annealings for 30 s, 70°C extensions for 30 s, a total of 40 cycles. The primer sequence was shown as follow.

circCUL2 F: 5′-AGAATACAGCAAGGGTGCAGA-3′ R: 5′-ACCACGGCTTTTATTGTCGT-3′ miR-208a-3p F: 5′-CGGGGCATA AGACGAGCAAAAA-3′ R: 5′-ATCCAGTGCAGGGTCCGAGG-3′ GAPDH F: 5′-GCACCGTCAAGGCTGAGAAC-3′ R: 5′-GGATCT CGCTCCTGGAAGATG-3′ U6 F: 5′-CTCGCTTCGG CAGCACA-3′ R: 5′-A ACGCTTCACGA ATTTGC GT-3′ PPP6C F: 5′-CCGCTGGATCTGGACAAGTAT-3′ R: 5′-ACACTGGCTGAACATTCGACT-3′.

### Immunofluorescence

Put the climbing piece into the 6-well plate, inseed the cells in the 6-well plate, and culture in the incubator for 24 hours, add the primer antibody. After incubating overnight, avoid the light and add the second antibody, DAPI re-staining nucleus. The distribution and number of LC3 fluorescence spots were detected under the fluorescence microscope.

### Western blot

The cells and tissues were added to the cell lysis buffer to make the cell cleavage. After the protein was collected, the protein concentration was detected by the BCA standard curve. After determining the protein concentration, each sample was boiled with a human 5x sample buffer to boil 5 min. The sodium dodecyl sulfate-polyacrylamide gel electrophoresis (SDS-PAGE) was performed. Then, the protein was transferred to the NC membrane, and then the NC membrane was sealed with 5% skim milk for 2 hours, the primary and second antibodies were incubated with specific antibodies, and GAPDH was used as the control.

### Flow cytometry

The logarithmic growth phase cells of each group were collected and re-suspended after low-temperature centrifugation to make a single-cell suspension. 5 μl Annexin V-FITC and 5 μl PI were added and incubated at room temperature for 15 min. 400 μl Binding Buffer was added to detect the apoptosis of cells by flow cytometry.

### Statistical analysis

The data were analyzed by Graphpad7.0 statistical software, the measurement data were expressed by mean ± SEM, an independent sample *t*-test was used for comparison between the two groups, and One-Way ANOVA was used for comparison between multiple groups.
